# Detection of Gelatin Adulteration in Traditional Chinese Medicine: Analysis of Deer-Horn Glue by Rapid-Resolution Liquid Chromatography-Triple Quadrupole Mass Spectrometry

**DOI:** 10.1155/2015/259757

**Published:** 2015-10-04

**Authors:** Jia Chen, Xian-Long Cheng, Feng Wei, Qian-Qian Zhang, Ming-Hua Li, Shuang-Cheng Ma

**Affiliations:** National Institute for Food and Drug Control, State Food and Drug Administration, 2 Tiantan Xili, Beijing 100050, China

## Abstract

Simultaneous identification of donkey-hide gelatin and bovine-hide gelatin in deer-horn glue was established by rapid-resolution liquid chromatography-triple quadrupole mass spectrometry. Water containing 1% NH_4_HCO_3_ was used for sample dissolution and trypsin was used for hydrolysis of the gelatins. After separation by a SB-C18 reversed-phase analytical column, collagen marker peptides were detected by mass spectrometry in positive electrospray ionization mode with multiple reaction monitoring. The method was specific, precise and reliable, and suitable for detection of adulterants derived from donkey-hide gelatin and bovine-hide gelatin in deer-horn glue.

## 1. Introduction

Deer-horn glue (*Cervi Cornus Colla*) is a traditional Chinese medicine (TCM) that has been widely used in China for about 2000 years. It is a solid glue prepared from deer horn by decoction and concentration [1]. It is viewed as a nutritious, high-quality TCM, as indicated in “Shennong's Herbal,” and is predominantly used for treating kidney disorders and Qi deficiency. It is claimed that long-time consumption of deer-horn glue will nourish yin, replenish blood, and prolong life. Because of the high market price and an inability to satisfy demand, adulteration is common and the most widely practiced approach is to substitute and/or replace the authentic material with donkey- and bovine-hide gelatin.

It has long been difficult to control the quality of deer-horn glue because of the absence of appropriate quality assessment methods. The polymerase chain reaction method has been used in DNA analysis for collagen identification [2, 3], but the method is not suitable for gelatin identification because of the breakdown of gelatin DNA during sample processing. Literature research has revealed that proteomic methods have been proposed as alternative tools for the assessment of collagen species in gelatins [4] and mass spectrometry has been successfully applied to elucidate differences among homological gelatins [5]. In our work, the focus of research has been on method specificity for differentiation of homological gelatins. In our previous work [6, 7], for instance, tryptic peptides of gelatins were measured by ultrahigh performance liquid chromatography-quadrupole time-of-flight mass spectrometry (UPLC-QTOF-MS), and principal component analysis was used to classify donkey-hide gelatin, bovine-hide gelatin, and deer-horn glue. Thereafter, gelatins were analyzed by doubly charged selected ion monitoring (DCSIM) with tandem mass spectrometry (MS/MS) to aid in the identification of the gelatins. The possibility of detecting the target peptides in such gelatins with rapid-resolution liquid chromatography (RRLC) coupled to electrospray ionization- (ESI-) ion trap (IT) MS would be a useful development.

Generally, HPLC-QQQ MS/MS is a sensitive analytical method available for detection of the adulterants. As shown recently, high-pressure liquid chromatography-mass spectrometry (HPLC-MS) is a widely used technique for qualitative and quantitative analyses, combining the efficient separation capability of HPLC with the powerful structural capability of MS [8–19]. In addition, the MS method offers the potential for high sensitivity and selectivity through multiple reaction monitoring (MRM) without the need for baseline chromatographic separation of the target analytes [20–22].

In the present work, RRLC-QQQ-MS with MRM has been used for characterization of deer-horn glue and detection of gelatin adulteration. A fully validated method has been developed, permitting measurement of the collagen marker peptides in commercial samples of deer-horn glue adulterated with donkey-hide and bovine-hide gelatins.

## 2. Experimental

### 2.1. Materials and Reagents

Formic acid was purchased from Sigma-Aldrich (St. Louis, MO, USA) and HPLC-grade acetonitrile (MeCN) was purchased from Fisher Scientific (Pittsburgh, PA, USA). Ultrahigh-purity water was prepared using a Milli-Q water purification system (Millipore Corporation, Bedford, MA, USA). Trypsin (sequencing grade) was obtained from Promega (Madison, WI, USA). Syringe filters (0.22 *μ*m) were purchased from Millipore (Billerica, MA, USA). All other chemicals used were of analytical grade. All samples were collected by the National Institute for Food and Drug Control.

### 2.2. Sample Preparation

First, 100 mg of the gelatin was dissolved in 50 mL of a 1% NH_4_HCO_3 _solution (pH 8.0). Then 10 *μ*L of trypsin solution (1 mg/mL in 1% NH_4_HCO_3_, pH 8.0) was added to 100 *μ*L of the gelatin solution. The mixture was incubated at 37°C for 12 h. All gelatin samples were prepared in this way. The sources of the gelatin samples are shown in Table 1.

### 2.3. Chromatographic Separation and Mass Spectrometry

The RRLC analysis was performed using an Agilent 1200 LC system (Agilent, MA, USA). Chromatographic separation was performed on an Agilent Zorbax SB-C18 reversed-phase analytical column (100 mm × 2.1 mm; 1.8 *μ*m particle size) at a column temperature of 45°C. The sample injection volume was 5 *μ*L. The mobile phase consisted of 0.1% formic acid in water (eluent A) and acetonitrile (eluent B). Gradient elution was performed as follows: 0–25 min eluent B 5% → 20%; 25–40 min eluent B 20% → 50%. The flow rate was 0.3 mL·min^−1^.

Mass spectrometry experiments were performed with an ESI source in positive ion mode. The vaporizer temperature was maintained at 350°C. The temperature of the drying gas was set at 350°C. The flow rate of the drying gas and the pressure of the nebulizer gas were set at 6 L/min and 60 psi, respectively. In MRM scan mode, the precursor and product ions should be set. The intensity of the precursor ion should be higher after optimizing the fragmentation voltage and the intensity of the product ion should also be higher after collision energy (CE) optimization. After optimization, the voltages for fragmentation and the CE were recorded (Table 2). An Agilent ChemStation was used for instrument (Agilent 6410B series triple quadrupole MS system) control and data processing. This included definitive identification of metabolites using retention times and fragmentation transition matching. Chromatographic separation was achieved using identical conditions to those described above for IT-MS experiments [6, 7]. Gradient elution was performed as follows: 0–25 min eluent B 5% → 20%; 25–40 min eluent B 20% → 50%. The flow rate was 0.3 mL·min^−1^.

## 3. Results and Discussion

Method validation was performed according to the guidelines of the Chinese Pharmacopoeia (2010 edition) for TCM. The key performance parameters evaluated were selectivity, signal linearity, sensitivity, and repeatability.

### 3.1. Selectivity

**Figure 1:**
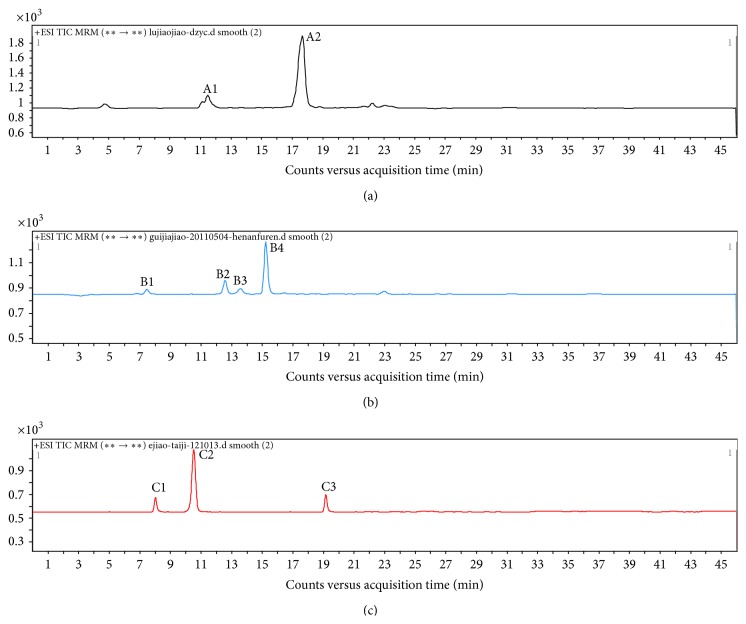
(a) Characteristic selected ion chromatograms for deer-horn glue. (b) Characteristic selected ion chromatograms for bovine-hide gelatin. (c) Characteristic selected ion chromatograms for donkey-hide gelatin.

The specificity of the method was investigated using deer-horn glue as a blank sample, while donkey- and bovine-hide gelatin serving as positive control samples. In previous work, the gelatins were characterized using DCSI-MS/MS. In this study, doubly charged ions at* m/z* 641.3, 747.5, 790.9, and 604.8, which are the species-specific peptides of bovine-hide gelatin, were selected for monitoring. Also, the fragments of these monitored ions resulted in the following additional characteristic molecular ion pairs:* m/z* 641.3 → 783.3, 641.3 → 726.2, 747.5 → 903.3, 747.5 → 847.1, 790.9 → 912.4, 790.9→841.3, 604.8→569.8, and 604.8→910.1. Doubly charged ions at 539.8, 618.8, and 765.9, which are species-specific peptides of donkey-hide gelatin, were selected for monitoring and yielded the following molecular ion transition pairs: 539.8 → 612.4, 539.8 → 923.8, 618.8 → 721.9, 618.8 → 778.9, 765.9 → 823.1, and 765.9 → 991.0. The chromatographic peaks were verified by checking the retention times and fragments of the peaks. As a result, chromatographic peaks for deer-horn glue were different to those of donkey-hide gelatin and bovine-hide gelatin. This meant that the mass spectra for the peptides in deer-horn glue were not subject to interference, as shown in Figure 1.

### 3.2. Signal Linearity

#### 3.2.1. Calibration Curves for Bovine-Hide Gelatin

A matrix solution of deer-horn gelatin standard was prepared by dissolving 100.0 mg of standard in 50 mL of a 1% NH_4_HCO_3_ solution (pH 8.0). Next, 100.6 mg of the bovine-hide gelatin standard was dissolved in 50 mL of a 1% NH_4_HCO_3_ solution (pH 8.0). Increasing aliquots (0.1, 0.5, 1.0, 1.5, and 5.0 mL) of the bovine-hide gelatin standard solutions were dissolved in 10 mL of the differing matrix solutions. Then, 100 *μ*L of the gelatin standard solution was taken and 10 *μ*L of trypsin solution (1 mg/mL in 1% NH_4_HCO_3_, pH 8.0) was added. The mixtures were incubated at 37°C for 12 h.

#### 3.2.2. Calibration Curves for Donkey-Hide Gelatin

For sample preparation, 119.6 mg of the donkey-hide gelatin standard was dissolved in 50 mL of a 1% NH_4_HCO_3_ solution (pH 8.0). This solution was then subjected to the same method as outlined in Section 3.2.1.

The regression equations, correlation coefficients, and test ranges for calibration are shown in Table 3. The results showed that there was an excellent correlation between the ratio of peak area response and concentration for each compound within the test ranges examined.

### 3.3. Sensitivity

The limit of detection (LOD), defined as the peak signal with a signal to noise ratio = 3/1, was determined based on injections (2 *μ*L) of low level standard solutions. The results demonstrated that the method was very sensitive with LODs of 10 × 10^−6 ^g/mL and 20 × 10^−6 ^g/mL for the peptides in the bovine- and donkey-hide gelatin samples, respectively.

### 3.4. Repeatability

**Figure 2:**
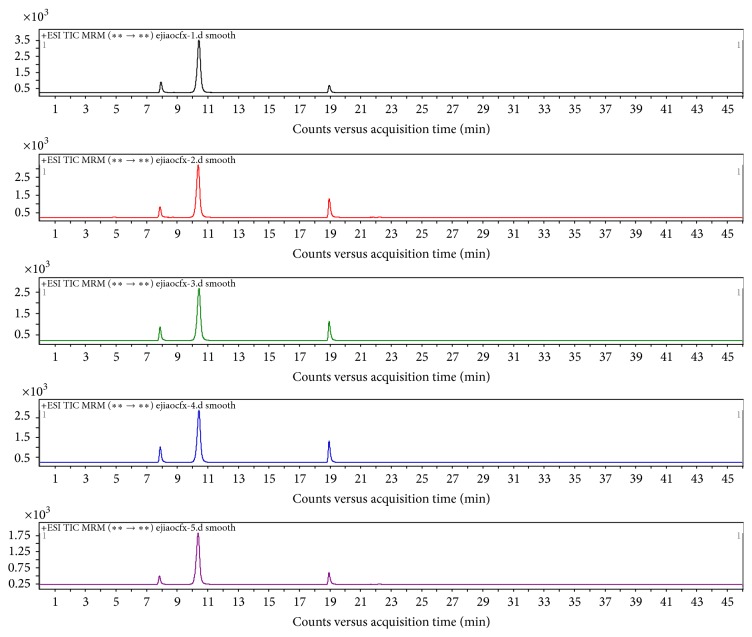
Characteristic selected ion chromatograms obtained for the tryptic digests of five donkey-hide gelatins.

**Figure 3:**
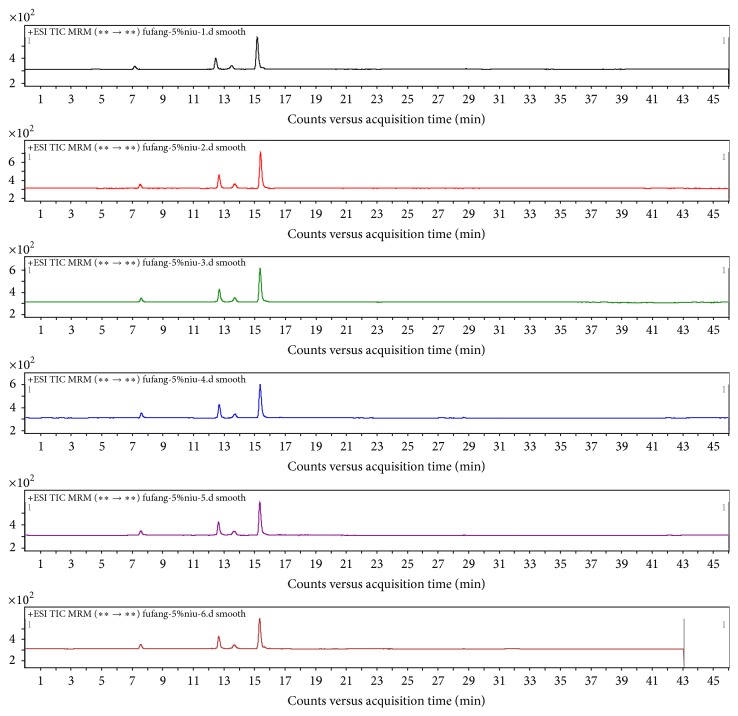
Characteristic selected ion chromatograms obtained for the tryptic digests of six bovine-hide gelatins.

Five replicate samples were prepared by the above method and the selected ion chromatograms, shown in Figures 2 and 3, confirm that the method provided reproducible detection of the collagen marker peptides.

### 3.5. Species Identification by RRLC-QQQ-MS

**Figure 4:**
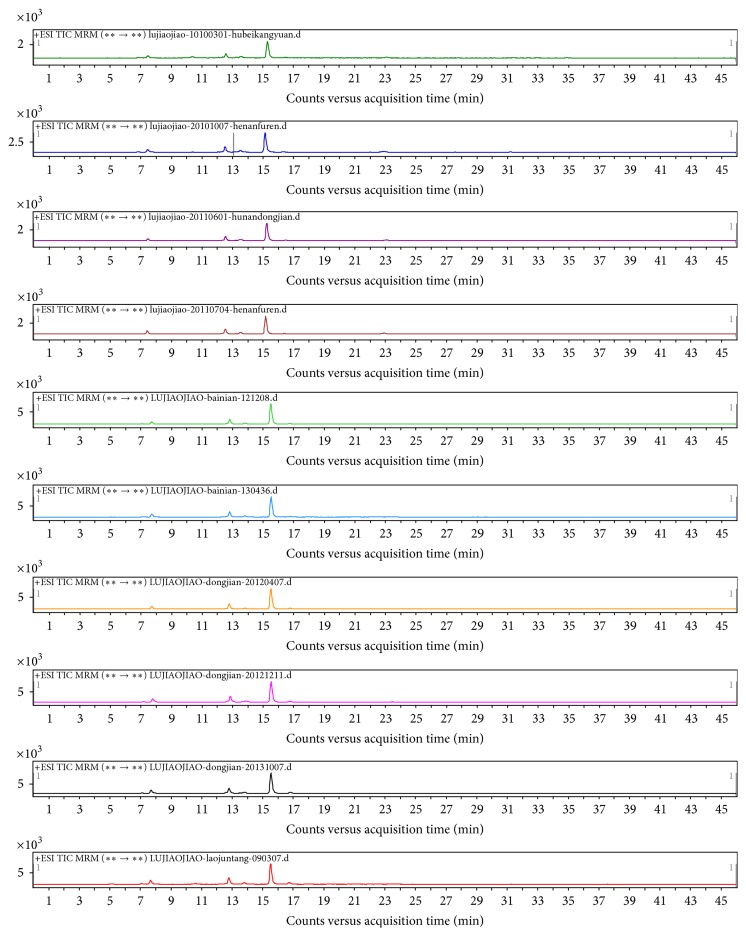
Representative MRM chromatograms for bovine-hide gelatins in tested samples.

**Figure 5:**
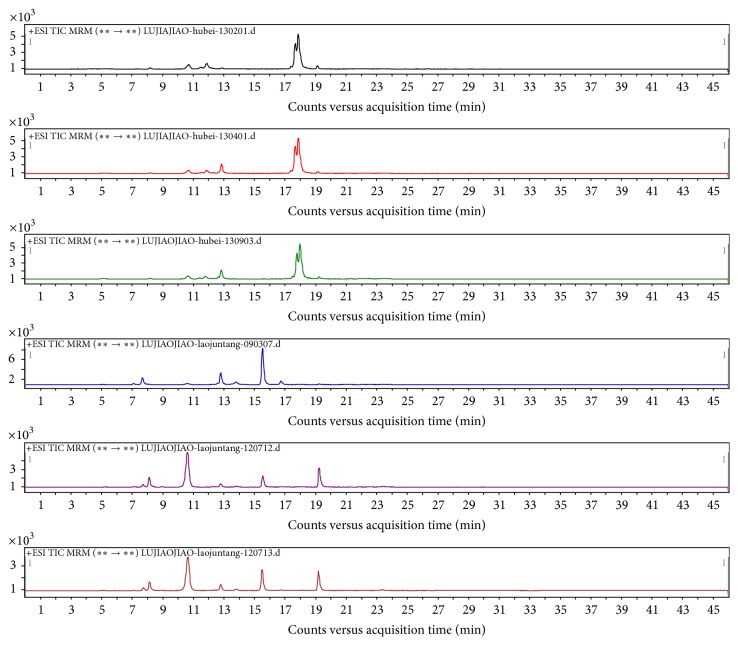
Representative MRM chromatograms for donkey-hide gelatins in tested samples.

The complex peptide pools obtained by tryptic digestion of the gelatins were subjected to LC-MS/MS and the characteristic molecular ion peaks for the bovine- and donkey-hide gelatins were detected as ion pairs listed in Table 2. Typical MRM chromatograms are shown in Figures 4 and 5. Commercial samples were positively identified after matching specific peptides in these samples with the corresponding reference samples. In 29 commercial samples of deer-horn glue analyzed, 12 tested positive for bovine-hide gelatin and 2 tested positive for donkey-hide gelatin, as indicated in Table 4. Overall, the proposed method provides a new and efficient route for unambiguous measurement of collagen marker peptides of bovine- and donkey-hide gelatins.

## 4. Conclusions

The RRLC-MS method with MRM provides an excellent qualitative tool for quality assessment of deer-horn glue because of its high sensitivity and specificity. As shown, collagen marker peptides associated with donkey-hide gelatin and bovine-hide gelatin and presented as adulterants in deer-horn glue, were readily detected. Furthermore, according to the signal linearity, we can estimate the amount of adulteration roughly and provide a specified limitation for adulteration. In survey analysis, almost 50% of commercial samples were found to have been adulterated by the addition of donkey- and/or bovine-hide gelatin, which were more than 3% of adulterants in samples according to the signal linearity.

## Figures and Tables

**Table 1:** Gelatin sources.

Sample	Standard gelatin	Source	Lot number by NIFDC
1	Deer-horn glue	*Cervus elaphus* Linnaeus	121694-201301
2	Donkey-hide gelatin	*Equus asinus* L.	121274-201202
3	Bovine-hide gelatin	*Bos taurusdomesticus* Gmelin	121695-201301

**Table 2:** Precursor and product ions for the gelatin species and operating parameters for fragmentation voltage and collision-activated dissociation voltage.

Number	Precursor	Product ion	Originated from	Retention time	Fragment voltage	Collision energy
	*m*/*z*	*m*/*z*		min	eV	eV
A1	732.8	817.9/961.9	Deer-horn glue	11.2080	175	30
A2	765.4	554.0/733.0	Deer-horn glue	17.1209	135	15
B1	641.3	783.3/726.2	Bovine-hide gelatin	7.4309	135	37
B2	790.9	912.4/841.3	Bovine-hide gelatin	12.5446	175	32
B3	747.3	903.3/847.1	Bovine-hide gelatin	13.4004	155	26
B4	604.8	569.8/910.1	Bovine-hide gelatin	15.2002	135	25
C1	618.8	721.9/778.9	Donkey-hide gelatin	7.7407	135	23
C2	539.8	612.4/923.8	Donkey-hide gelatin	10.1043	135	15
C3	765.9	823.1/991.0	Donkey-hide gelatin	18.8379	155	45

**Table 3:** Signal linearity curves for two analytes.

Analytes	Linear equations	Range (*μ*g/mL)	*R* ^2^
Bovine-hide gelatin	*Y* = 3715*X* + 321.1	20.12–1006	0.957
Donkey-hide gelatin	*Y* = 32485*X* − 1130	23.92–1196	0.995

**Table 4:** Results for commercial samples of deer-horn glue.

Number	Sample	Number	Origin	Donkey-hide gelatin	Bovine-hide gelatin	Deer-horn glue
1	Deer-horn glue	001	Henan Province	−	+	+
2	Deer-horn glue	002	Henan Province	−	+	−
3	Deer-horn glue	003	Shandong Province	−	+	−
4	Deer-horn glue	004	Henan Province	−	+	+
5	Deer-horn glue	005	Hubei Province	−	+	−
6	Deer-horn glue	006	Hunan Province	−	+	+
7	Deer-horn glue	007	Hebei Province	+	−	+
8	Deer-horn glue	008	Hebei Province	+	−	+
9	Deer-horn glue	009	Hunan Province	−	+	+
10	Deer-horn glue	010	Henan Province	−	+	+
11	Deer-horn glue	011	Henan Province	−	+	−
12	Deer-horn glue	012	Hunan Province	−	+	+
13	Deer-horn glue	013	Inner Mongolia Autonomous Region	−	+	+
14	Deer-horn glue	014	Shandong Province	−	+	+
15	Deer-horn glue	015	Shandong Province	−	−	+
16	Deer-horn glue	016	Shandong Province	−	−	+
17	Deer-horn glue	017	Beijing Municipality	−	−	+
18	Deer-horn glue	018	Beijing Municipality	−	−	+
19	Deer-horn glue	019	Beijing Municipality	−	−	+
20	Deer-horn glue	020	Hubei Province	−	−	+
21	Deer-horn glue	021	Hubei Province	−	−	+
22	Deer-horn glue	022	Hubei Province	−	−	+
23	Deer-horn glue	023	Henan Province	−	−	+
24	Deer-horn glue	024	Henan Province	−	−	+
25	Deer-horn glue	025	Henan Province	−	−	+
26	Deer-horn glue	026	Shandong Province	−	−	+
27	Deer-horn glue	027	Shandong Province	−	−	+
28	Deer-horn glue	028	Beijing Municipality	−	−	+
29	Deer-horn glue	029	Beijing Municipality	−	−	+
30	Deer-horn glue	121694-201301	Standard gelatin from NIFDC	−	−	+
31	Donkey-hide gelatin	121274-201202	Standard gelatin from NIFDC	+	−	−
32	Bovine-hide gelatin	121695-201301	Standard gelatin from NIFDC	−	+	−
